# POLYPHARMACY: TRANSLATIONAL RESEARCH TO UNDERSTAND EFFECTS OF AGE, SEX, AND FRAILTY ON KEY OUTCOMES FOR FORTITUDE

**DOI:** 10.1093/geroni/igae098.1646

**Published:** 2024-12-31

**Authors:** John Mach

**Affiliations:** Kolling Institute/The University of Sydney/Royal North Shore Hospital, Sydney, New South Wales, Australia

## Abstract

Chronic medication use is common in older men and women. Older people, particularly those with polypharmacy (use of 5 or more drugs) for multi-morbidity, are rarely included in clinical trials to establish efficacy and safety of therapeutic drugs. Based on clinical observational studies, polypharmacy is associated with impaired physical function and exacerbates frailty in older adults, particularly women, who make up the majority of this age group. In addition, there is evidence that women are at greater risk of drug-related harm than men. Preclinical models would be useful to screen for adverse geriatric outcomes and understand the impact of age, sex and frailty. This presentation will cover our advancements in the development and use of preclinical models to understand the effects of polypharmacy on geriatric outcomes that are important contributors to the ‘fortitude factor’ in both sexes and old age. We will present recent data from studies in our preclinical models that: (1) describe how chronic polypharmacy use impairs translatable functional outcomes in ageing; (2) demonstrate differences in outcome response for different sex, age and frailty.

